# Superficial venous thrombosis as a possible consequence of ChAdOx1 nCoV-19 vaccine: two case reports

**DOI:** 10.1186/s13256-022-03407-6

**Published:** 2022-05-07

**Authors:** Mukesh Kumar Sah, Bishnu Mohan Singh, Puja Sinha, Prerit Devkota, Sudhira Kumari Yadav, John Shrestha, Ashis Shrestha

**Affiliations:** 1 Department of Emergency Medicine and General Practice, Patan Academy of Health Sciences, Lalitpur, Bagmati Province Nepal; 2 Patan Hospital, Lalitpur, Nepal; 3 Public Health Expert, Louisiana State University, Shreveport, LA USA; 4 Department of Emergency Medicine and General Practice, Patan Academy of Health Sciences, Lalitpur, Nepal

**Keywords:** ChAdOx1 nCoV-19, Covishield vaccine, Superficial venous thrombosis (SVT), Venous thrombosis, Superficial saphenous vein (SSV), COVID 19

## Abstract

**Background:**

Many scientists across the world got involved in the race to develop successful anti-SARS-CoV-2 vaccines to overcome COVID-19 pandemic. Among the different vaccines developed against SARS-CoV-2, Covishield was the first vaccine approved for emergency use in Nepal. We report two cases of Superficial Vein Thrombosis (SVT) for the first time in the literature after vaccination with the Chimpanzee Adenovirus-vectored Vaccine (ChAdOx1 nCoV-19 vaccine).

**Cases presentation:**

Two cases, a 24-year-old young Chhetri male and a 62-year-old Chhetri female who have received Covishield (ChAdOx1 nCoV-19) vaccine, developed pain in left calf after 2 weeks and 10 weeks of vaccination, respectively. Both the case belongs to the Chhetri ethnic group of Nepal. The pain became severe on the fourth week of immunization in the first case while the pain was acute and severe on the 10^th^ week of vaccination in the second case. The first presented to emergency room and second case was referred to the emergency room from Orthopedic Clinic. On evaluation the first patient had normal vitals with no history of fever and swelling yet displayed non-radiating mild to moderate intensity pain localized to left leg below the knee which became aggravated by movements. In the second case however pain was more intense with other characteristics as first case. Both cases had low wells score (< 4). On local examination tenderness was noted on squeezing but other systemic examination findings of the patient were within normal limits in both cases. Among the numerous vaccines used to fight the battle against COVID-19 disease, the ChAdOx1 nCoV-19 vaccine, Covishield, has been widely used in Nepal and India. Apart from other minor side effects, in few cases thromboses have been reported after vaccination of ChAdOx1 nCoV-19, Covishield, vaccine.

**Conclusion:**

These cases reporting Superficial Vein Thrombosis may be an additional adverse effect to the list of adverse events associated with ChAdOx1 nCoV-19, Covishield, vaccine. However, the benefits of the vaccine in breaking the chain of COVID 19 spread are certainly greater than the risk of thromboses.

## Highlights/Take-away message

1. The Chimpanzee Adenovirus-vectored Vaccine (ChAdOx1 nCoV-19 vaccine) has been widely used in Himalayan Country, Nepal and India.
2. Apart from other minor side effects, delayed thromboses in superficial vein have been reported after vaccination of ChAdOx1 nCoV-19 vaccine and they may be the consequences of vaccination though larger study is needed to verify them.
3. Superficial vein thrombosis (SVT) should be an additional adverse effect to be mentioned in the list of adverse events associated with ChAdOx1 nCoV-19, Covishield, vaccine.
4. The benefits of the vaccine in breaking the chain of COVID 19 spread are certainly greater than the risk of thromboses.

## Background

Since its first identification in December 2019 in Wuhan, the Severe Acute Respiratory Syndrome-Corona Virus-Type 2 (SARS-CoV-2) virus has spread globally causing the era of COVID 19 pandemic [1]. SARS-CoV-2 outbreak has caused detrimental effects not only on the global healthcare systems but also on social and economic aspect of human life [2]. The pandemic has affected various sectors including agriculture, manufacturing industry, education and finance industry, tourism and so on [2]. As vaccination is one of the most effective response to such outbreak, many scientists across the world got involved in the race to develop successful anti-SARS-CoV-2 vaccines. Among the different vaccines developed against SARS-CoV-2, Covishield was the first vaccine approved for emergency use in Nepal. Covishield, also known as ChAdOx1 nCoV-19 vaccine (AZD1222), is a replication-deficient chimpanzee adenoviral vector vaccine that contains SARS-CoV-2 structural surface glycoprotein antigen gene and was developed at Oxford University [3]. The different clinical trials that were conducted to evaluate the safety and efficacy of Covishield showed that the vaccine was safe and efficacious in the trial population [3, 4]. Nepal started its first phase vaccination drive starting on Jan 27, 2021, with the COVISHIELD vaccine for frontline health workers [5]. According to a cross-sectional study conducted at Patan Academy of Health Sciences (PAHS), Nepal to calculate the incidence of Adverse Events Following Immunization (AEFI) observed after the first dose of Covishield vaccine minor AEFIs were seen in 85.1 % of recipients whereas serious AEFI and severe minor AEFI were in 0.06 % and 0.03% recipients, respectively [5]. In this study, one case of anaphylaxis was observed, however no deaths were reported [5]. In the EudraVigilance drug safety database there has been reported 62 cases of cerebral venous sinus thrombosis, 24 cases of spanchnic vein thrombosis, 18 cases of the fatal thrombotic events among 25 million recipients of the ChAdOx1 nCov-19 vaccine [6]. There have been 2 reported cases of deep vein thrombosis and one case of bilateral superior ophthalmic vein thrombosis after ChAdOx1 nCoV-19 vaccination [7, 8]. Here in this article, we report two cases of Superficial Vein Thrombosis (SVT) for the first time in the literature after vaccination with the ChAdOx1 nCoV-19 vaccine.

## Method

This case report has been prepared following the CARE guidelines, the guidelines developed to increase the accuracy, transparency and usefulness of case reports.

## Case presentations

### First case presentation

A 24-year-old young male who belongs to the Chhetri ethnic group of Nepal received the Covishield vaccine and developed pain in the left calf after 2 weeks. Initially, the pain was mild but later on the intensity of the pain gradually increased and it became severe on the fourth week of immunization. This case presented to emergency department on April 4, 2021. On evaluation, the patient complained of mild to moderate intensity pain that was non-radiating localized to left leg below the knee and aggravated by movements. The patient did not present any history of fever or swelling of the leg. The patient had no history of trauma to the leg in the area of pain. He had no similar presentation in the past. He did not use to smoke. He was living with his parents. He was a student. His family is middle income family. The patient was taking acetaminophen as analgesic. On vitals assessment, his pulse was 64 beats/min, blood pressure was 110/80 mm of Hg, respiratory rate 16 breaths/min and oxygen saturation was 97%. On local examination there was neither swelling of the left calf nor local temperature was raised. On squeezing the left calf, tenderness was noted. Wells score was low (< 4). Other systemic examination findings of the patient were within normal limits.

#### Relevant investigation

Complete Blood Count: white cell count (WBC) 6100/μL; neutrophils (N) 60%; lymphocytes (L) 38%; eosinophil (E) 2%; red blood cells (RBC) 5.1 × 10^12^/L; haemoglobin 14.5 g/dL; Hematocrit 45%; platelets 337,000/﻿μL.

PT 13.8 sec (Control 12 Sec); INR 0.98.

Lipid Profile: Cholesterol Total 114 mg/dL; Triglyceride 68 mg/dL; High-Density lipoprotein (HDL) cholesterol 48 mg/dL; Low-Density Lipoprotein (LDL) cholesterol 52 mg/dL.

Biochemistry: Urea 23 mg/dL; Creatinine 1 mg/dL; Sodium 139 mMol/L; Potasium 4.5 mMol/L;

Electrocardiogram (ECG) changes: Sinus Rhythm (Regular), Rate 64 bpm.

Hepetitis B Surface Antigen (HBsAg) non reactive; Hepatitis C Virus Antibody (HCVAb) non reactive; Human Immunodeficiency Virus Antibody (HIVAb) non reactive.

Venous Doppler USG left leg:

Report of April 4, 2021.

Superficial Thrombophlebitis of Superficial Saphenous Vein (SSV).

Details: Echogenic thrombus causing complete narrowing of proximal 1/3rd of SSV was noted, about 1.41 cm from Sapheno Popliteal Junction (SPJ). Circumferential mural thickening of distal 2/3^rd^ of SSV where slow flow is noted.

Report of April 8, 2021.

Superficial Thrombophlebitis of SSV.

**Fig. 1:**
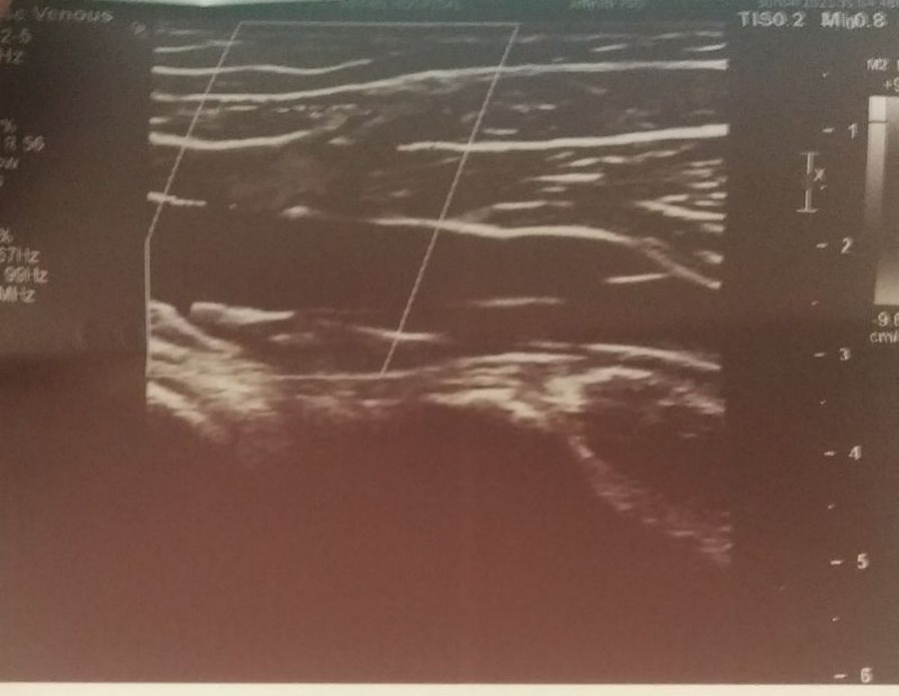
Doppler of proximal end of Superficial Saphenous Vein

**Fig. 2:**
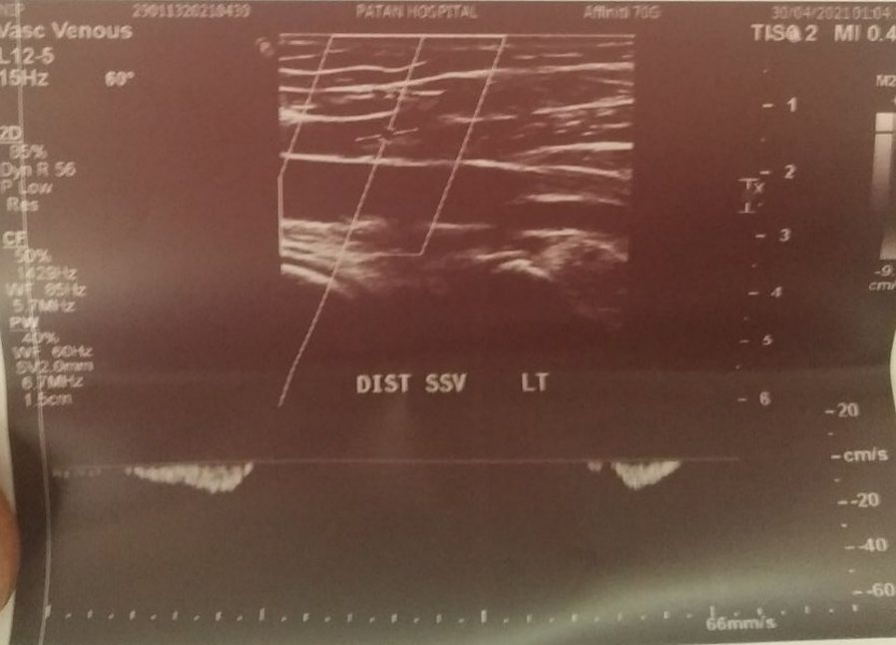
Doppler of distal end of Superficial Saphenous Vein

Details: Echogenic thrombus causing complete narrowing of proximal 1/3rd of SSV was noted, with almost similar findings in previous Doppler report. The thickening of the proximal part of SSV was 3.5 mm in diameter (Fig. 1) whereas that of the distal part of SSV was 2.4 mm in diameter (Fig. 2).

Bed side renal Doppler at Emergency Room:

Renal Arterial Resistance Index (RI): 0.6 (right kidney) and 0.56 (left kidney).

Bed side Echocardiography at Emergency Room:

LVEF: 60%; No other gross abnormalities were noted.

#### Differential diagnosis

Our provisional diagnosis was superficial venous thrombosis (SVT). The main differential diagnoses were deep vein thrombosis (DVT) and cellulitis. The patient did not have fever and skin discoloration at the pain site which could easily rule out cellulitis. But we could differentiate between DVT and SVT via ultrasonic examination of the left lower leg that showed superficial chronic thrombotic changes of the left Small Saphenous Vein (SSV).

#### Treatment

The patient was prescribed acetaminophen as analgesic and topical heparinized cream as anticoagulant. No systemic anticoagulant was prescribed.

#### Outcome and follow-up

The pain subsided and there was much clinical improvement in the symptoms in the last 4 weeks after using heparinized topical cream.

#### Patient perspective

Patient felt good with the treatment received as it was non invasive and no admission was required.

### Second case presentation

A 62-year-old young female who also belongs to the Chhetri ethnic group of Nepal received Covishield vaccine and developed pain in left calf after 10 weeks of vaccination. Contrary to the former case, her pain was severe from the beginning. The patient presented to orthopedics OPD for her leg pain and was referred to emergency department on May 03, 2021. She had co-morbidities of Hypertension, Hypothyroidism and Bronchial Asthma. She was taking Losartan for Hypertension, Thyroxine Sodium for Hypothyroidsim and occasional MDI Asthalin for Bronchial Asthma. She has one son and one daughter. She was a non smoker and never consumed alcohol. Her family was middle income family. She was a teacher by profession. On evaluation, the patient complained of pain of severe intensity that was non-radiating localized to the left leg below the knee and aggravated by movements. There was neither history of fever nor swelling of the leg. The patient did not have any history of trauma to the leg at the site of pain. The patient was taking acetaminophen as an analgesic on the first day. On initial assessment, her pulse was 78 beats/min, blood pressure was 130/80 mmHg, respiratory rate 18 breaths/min and oxygen saturation was 96%. Similar to previous case, local examination of this patient showed no swelling of left calf and no increase in the local temperature. In addition, tenderness was also noted upon compression of the left calf. Wells score was low (< 4) as observed in first patient. Other systemic examination findings of the patient were within normal limits in this case as well.

#### Relevant investigation of the second

Complete Blood Count: white cell count (WBC) 8000/μL; neutrophils (N) 70%; lymphocytes (L) 27%; monocyte (M) 2%, eosinophil (E) 1%; red blood cells (RBC) 4.5 × 10^12^/L; haemoglobin 14.2 g/dL; Hematocrit 42%; platelets 245,000/μL.

APTT 19 Sec; PT 13.6 sec (Control 12 Sec); INR 0.97.

CRP = 1 mg/L.

Venous Doppler USG left leg:

Report of Date: May 03, 2021.

Superficial Chronic thrombotic Changes of SSV.

Details: Acute thrombus partially occluding lumen of proximal SSV. SSV distal thrombus could not be assessed.

#### Differential diagnosis

Our initial diagnosis was deep venous thrombosis (DVT). The main differential diagnoses were superficial vein thrombosis (SVT) and cellulitis. As in first case in this case also the patient did not have fever and skin discoloration at the pain site which could easily rule out cellulitis. But we could differentiate between DVT and SVT via ultrasonic examination of left lower leg that showed superficial thrombophlebitis of left Small Saphenous Vein (SSV).

#### Treatment

The patient was prescribed acetaminophen for pain relief and topical heparinized cream. In this case Tab Aspirin 75 mg was prescribe on daily basis but no anticoagulant was prescribed.

#### Outcome and follow-up

The pain subsided and there was much clinical improvement in the symptoms in the last 3 weeks after using heparinized topical cream and Tab Aspirin.

#### Patient perspective

Patient was concerned about pain and her pain was relieved after medications, and she felt good with the outcome of the treatment.

## Discussion

In these two cases, patients presented with pain over lower limb and the diagnosis of superficial venous thrombosis was made. Both had no significant risk factor for venous thrombosis however, the common factor in both was that they both had received ChAdOx1 nCoV-19 vaccine against COVID-19.

COVID 19 disease causes thrombosis risk which could be explained as a result of complex systemic inflammatory response, excessive coagulation status, blood stasis, and endothelial dysfunction following SARS-CoV-2 infection [9, 10]. There have been several cases of deep vein thrombosis (DVT) and superficial vein thrombosis (SVT) reported in the literature following COVID 19 infection. A case has been reported in literature where a COVID 19 patient developed SVT of Great Saphenous Vein (GSV) [9]. The patient had no risk factors for SVT, but he was diagnosed with SVT of GSV of the right leg via ultrasound examination [9]. In our case both patients tested negative on both RT-PCR and COVID 19 antigen tests.

Various cases of thrombosis have been reported in literature after ChAdOx1 nCoV-19 vaccination. Two vaccine recipients developed deep vein thrombosis (DVT) at 27 and 29 days following vaccination [8]. These two cases had a negative d-dimer test and a low Wells score but were diagnosed with DVT. Similarly in our cases the patients had no swelling or skin discoloration at the pain site but we could diagnose it SVT of left SSV with the help of ultrasound examination. There has been a case reported in literature where a 55-year-old woman developed bilateral superior ophthalmic vein thrombosis after ChAdOx1 nCoV-19 vaccination [8]. In a case series that studied 11 patients in Germany and Austria, 9 patients had cerebral venous thromboses, 3 had splanchnic-vein thrombosis, 3 had pulmonary embolism and 4 had other thromboses like Aortoiliac, right intraventricular, iliofemoral vein, widespread microvascular thrombi, multiple organ thrombi [11]. The authors of this case series also clearly pointed out that immune thrombotic thrombocytopenia mediated by platelet-activating antibodies against platelet factor 4 (PF4) is responsible for clot formation after vaccination [11]. This new phenomenon of thromboses after ChAdOx1 nCoV-19 vaccination is termed as vaccine induced prothrombotic immune thrombocytopenia (VIPIT) [12]. Thrombocytopenia was however not seen in both of our cases.

Nevertheless, we hypothesized that process of thrombosis could have started much earlier than the symptom of pain developed. Therefore there is a high possibility that this may be a delayed consequence of vaccination of ChAdOx1 nCoV-19 vaccine. This phenomenon of thrombus formation may have developed in many cases but mild pain may have been ignored by many patients and they did not appear in the hospital. Though we found two such cases, to verify this statement, a study among large population should be done to verify whether this is rare complication or a common adverse event that is frequently missed.

## Conclusion

There have been different vaccines on the race to fight the battle against COVID 19 disease. The ChAdOx1 nCoV-19 vaccine has been widely used in Nepal and India. These two cases of superficial venous thrombosis may be an addition to the list of adverse events associated with ChAdOx1 nCoV-19, vaccine. However, the benefits of the vaccine in breaking the chain of COVID 19 spread far outweigh, the risk of thromboses.

## Data Availability

All information including reports will be available from author and patient, if required.

## Abbreviations

AEFI: Adverse effect following immunization
ChAdOx1 nCoV-19: Chimpanzee adenovirus-vectored vaccine
COVID: Corona virus disease
ECG: Electrocardiogram
GSV: Great sephanous vein
LVEF: Left ventricular ejection fraction
PAHS: Patan Academy of Health Sciences, Lagankhel, Lalitpur
SARS-CoV-2: Severe acute respiratory syndrome-corona virus-type 2
SPJ: Saphenopopleteal junction
SSV: Superficial sephanous vein
SVT: Superficial vein thrombosis
VIPIT: Vaccine induced prothrombotic immune thrombocytopenia
