# Correction: Superficial venous thrombosis as a possible consequence of ChAdOx1 nCoV-19 vaccine: two case reports

**DOI:** 10.1186/s13256-022-03495-4

**Published:** 2022-06-27

**Authors:** Mukesh Kumar Sah, Bishnu Mohan Singh, Puja Sinha, Prerit Devkota, Sudhira Kumari Yadav, John Shrestha, Ashis Shrestha

**Affiliations:** 1 Department of Emergency Medicine and General Practice, Patan Academy of Health Sciences, Lalitpur, Bagmati Province Nepal; 2 Patan Hospital, Lalitpur, Nepal; 3 Public Health Expert, Louisiana State University, Shreveport, LA USA; 4 Department of Emergency Medicine and General Practice, Patan Academy of Health Sciences, Lalitpur, Nepal

## Correction: Journal of Medical Case Reports (2022) 16:182 https://doi.org/10.1186/s13256-022-03407-6

Following publication of the original article [1], the authors reported an error in the title.

The incorrect title is:

- Superficial venous thrombosisas a possible consequence of ChAdOx1 nCoV-19 vaccine: two case reports.

The correct title is:

- Superficial venous thrombosis as a possible consequence of ChAdOx1 nCoV-19 vaccine: two case reports.

The title has been updated above and the original article [1] has been corrected.
